# Identification of the pyroptosis‑related prognostic gene signature and the associated regulation axis in lung adenocarcinoma

**DOI:** 10.1038/s41420-021-00557-2

**Published:** 2021-06-25

**Authors:** Wanli Lin, Ying Chen, Bomeng Wu, Ying chen, Zuwei Li

**Affiliations:** Department of thoracic surgery, Gaozhou people’s hospital, Gaozhou, 525200 China

**Keywords:** Non-small-cell lung cancer, Prognostic markers

## Abstract

Lung adenocarcinoma (LUAD) remains the most common deadly disease and has a poor prognosis. Pyroptosis could regulate tumour cell proliferation, invasion, and metastasis, thereby affecting the prognosis of cancer patients. However, the role of pyroptosis-related genes (PRGs) in LUAD remains unclear. In our study, comprehensive bioinformatics analysis was performed to construct a prognostic gene model and ceRNA network. The correlations between PRGs and tumour-immune infiltration, tumour mutation burden, and microsatellite instability were evaluated using Pearson’s correlation analysis. A total of 23 PRGs were upregulated or downregulated in LUAD. The genetic mutation variation landscape of PRG in LUAD was also summarised. Functional enrichment analysis revealed that these 33 PRGs were mainly involved in pyroptosis, the NOD-like receptor signalling pathway, and the Toll-like receptor signalling pathway. Prognosis analysis indicated a poor survival rate in LUAD patients with low expression of NLRP7, NLRP1, NLRP2, and NOD1 and high CASP6 expression. A prognostic PRG model constructed using the above five prognostic genes could predict the overall survival of LUAD patients with medium-to-high accuracy. Significant correlation was observed between prognostic PRGs and immune-cell infiltration, tumour mutation burden, and microsatellite instability. A ceRNA network was constructed to identify a lncRNA KCNQ1OT1/miR-335-5p/NLRP1/NLRP7 regulatory axis in LUAD. In conclusion, we performed a comprehensive bioinformatics analysis and identified a prognostic PRG signature containing five genes (NLRP7, NLRP1, NLRP2, NOD1, and CASP6) for LUAD patients. Our results also identified a lncRNA KCNQ1OT1/miR-335-5p/NLRP1/NLRP7 regulatory axis, which may also play an important role in the progression of LUAD. Further study needs to be conducted to verify this result.

## Introduction

Lung cancer remains the most common deadly disease, with an estimated 2.09 million new cases and 1.76 million deaths each year [1]. Worse still, the incidence and mortality of lung cancer are rising [1]. Lung adenocarcinoma (LUAD) is the most common histologic subtype of lung cancer, accounting for approximately 40% of all cases [2]. Despite surgery, chemoradiotherapy, targeted therapy, and immunotherapy being used in the treatment of lung cancer, the prognosis remains disheartening [3], and 5-year survival ranges from 4 to 17%, depending on disease and treatment differences [4].

Although many biomarkers or gene signatures have been found to have the potential to predict the prognosis of LUAD, they are still in the molecular research phase and have not yet been applied in clinical practice. Thus, uncovering prognostic gene signatures for the prognosis of LUAD would be of great significance.

Pyroptosis, referred to as cellular inflammatory necrosis, is considered to be gasdermin-mediated programmed necrotic cell death [5]. Triggered by certain inflammasomes, pyroptosis relies on the cleavage of gasdermin D (GSDMD) and activation of inactive cytokines [6]. The correlation between pyroptosis and cancer is extremely complicated. Although pyroptosis can inhibit the oncogenesis and progression of tumours, it also develops a microenvironment delivering nutrients for cancer and accelerating cancer growth [7]. Increasing studies have demonstrated the effect of pyroptosis on tumour cell proliferation, invasion, and metastasis, thus affecting the prognosis of cancer [8, 9]. For example, a recent study identified a novel pyroptosis-related gene signature for the prognosis of ovarian cancer [10]. In lung cancer, the pyroptosis gene GSDMD can inhibit tumour proliferation by regulating the intrinsic mitochondrial apoptotic pathway and EGFR/Akt signalling [11]. The prognostic value of pyroptosis-related genes (PRGs) in LUAD has not yet been elucidated.

In the current research, bioinformatics analysis was performed to investigate PRG expression profiles and their prognostic significance as well as the associated regulatory axis in LUAD. Our data may provide additional evidence for prognostic biomarkers and therapeutic targets for LUAD.

## Results

### Defining of the expression of PRGs in LUAD

**Fig. 1:**
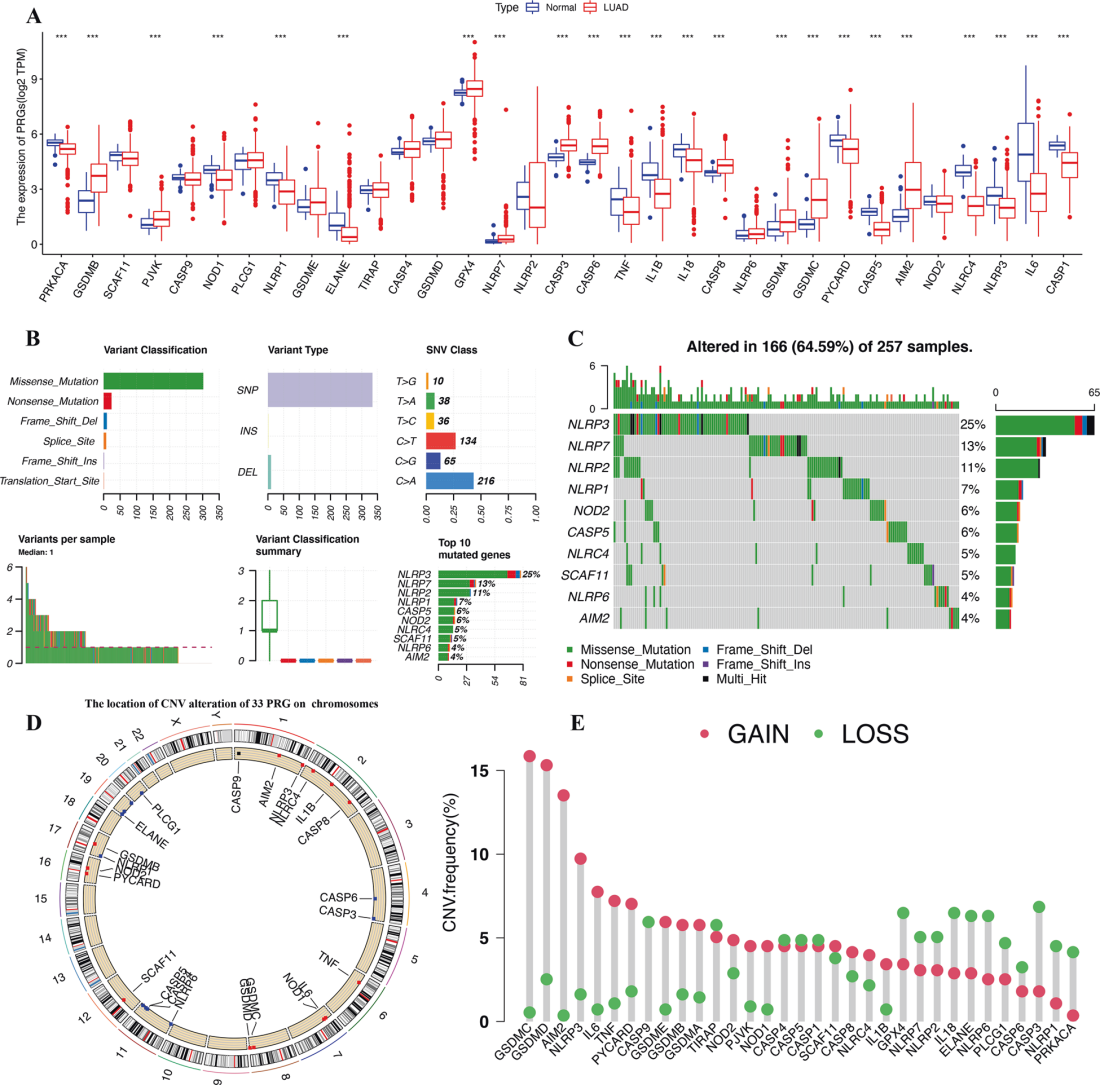
Landscape of genetic and expression variation of PRG in LUAD. **A** The expression of 33 PRG in LUAD and lung tissues, Tumour, red; Normal, blue. The upper and lower ends of the boxes represented the interquartile range of values. The lines in the boxes represented median value. **B, C** The mutation frequency and classification of 33 PRG in LUAD. **D** The location of CNV alteration of 33 PRG on 23 chromosomes in the LUAD cohort. **E** The CNV variation frequency of 33 PRG in the LUAD cohort. The height of the column represented the alteration frequency. ***P < 0.001, PRG pyroptosis‑related gene, LUAD lung adenocarcinoma, SNP single nucleotide polymorphism, INS insertion, DEL deletion.

We first explored the expression of the 33 PRGs in LUAD and normal lung tissues using the TCGA LUAD dataset. A total of 23 PRGs were either upregulated or downregulated in LUAD (Fig. 1A). More specifically, the expression of *PRKACA*, *NOD1*, *NLRP1*, *ELANE*, *TNF*, *IL1B*, *IL18*, *PYCARD*, *CASP5*, *NLRC4*, *NLRP3*, *IL6*, and *CASP1* was increased, while the expression of *GSDMB*, *PJVK*, *CASP4*, *NLRP7*, *CASP3*, *CASP6*, *CASP8*, *GSDMA*, *GSDMC*, and *AIM2* was decreased in LUAD compared with normal tissues (Fig. 1A, all <0.001). A protein–protein interaction (PPI) analysis with the minimum required interaction score of 0.9 was constructed to detect the interactions of these PRGs, which revealed that *CASP1*, *CASP5*, *CASP8*, *NLRP1*, *NLRP3*, and *PYCARD* were hub genes (Fig. S1A). Supplementary Fig. S1B shows the correlation network containing all PRGs.

### Landscape of genetic variation of PRGs in LUAD

We then summarised the incidence of copy number variations and somatic mutations of 33 PRGs in LUAD. As shown in Fig. 1B and Fig. 1C, 116 of 257 (64.59%) LUAD samples demonstrated genetic mutations. Missense mutation was the most common variant classification (Fig. 1B). SNPs were the most common variant type, and C > A ranked as the top SNV class. The results also demonstrated *NLRP3* as the gene with the highest mutation frequency, followed by *NLRP7* and *NLRP2*, among the 33 PRGs (Fig. 1C). Figure 1D presents the location of CNV alterations of these 33 PRGs on chromosomes. We also investigated CNV alteration frequency, which revealed that these 33 PRGs showed prevalent CNV alterations. More than half of the 33 PRGs had copy number amplification, while the CNV deletion frequencies of *CASP9*, *GPX4*, *NLRP7*, *NLRP2*, *IL18*, *ELANE*, *NLRP6*, *PLCG1*, *CASP6*, *CASP3*, *NLRP1*, and *PRKACA* were widespread (Fig. 1E).

### Functional enrichment analysis of PRGs

**Fig. 2:**
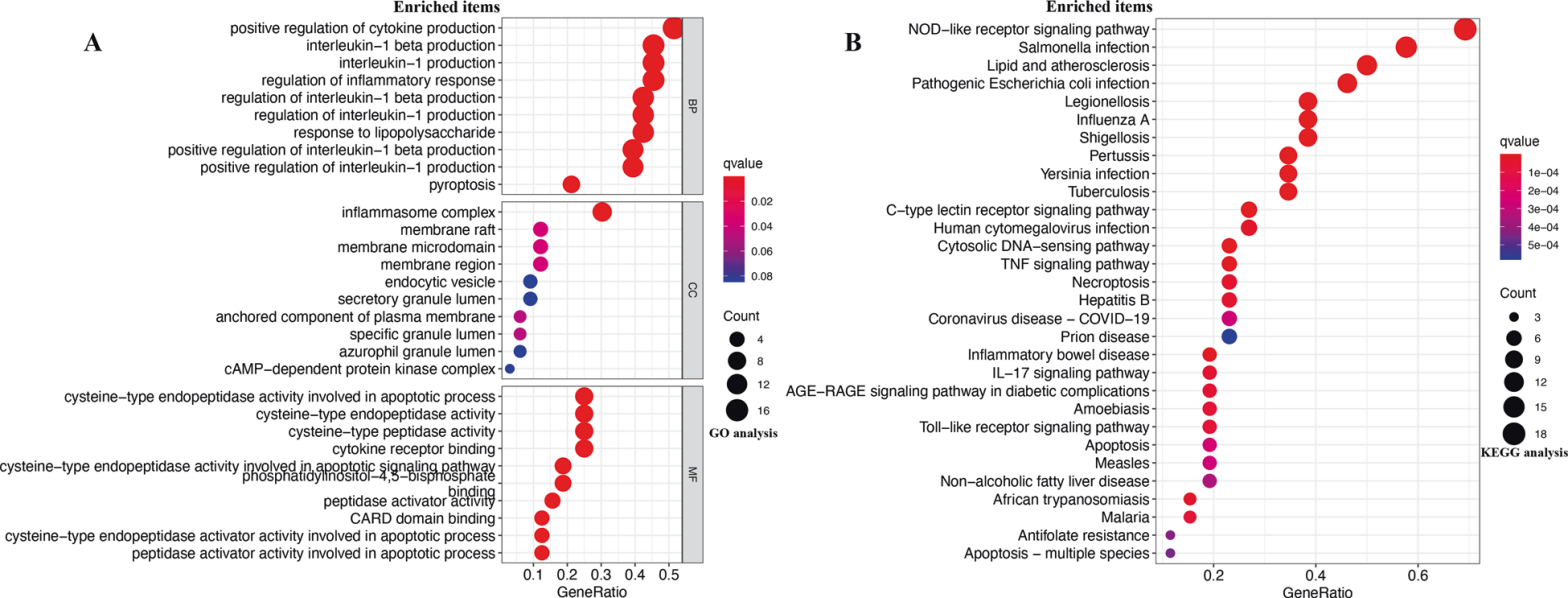
The functional enrichment analysis of PRG in LUAD. **A** The enriched item in gene ontology analysis. **B** The enriched item in Kyoto Encyclopedia of Genes and Genomes analysis. The size of circles represented the number of genes enriched. BP biological process, CC cellular component, MF molecular function, PRG pyroptosis‑related gene.

To clarify the function of PRGs, the pathways were analysed using GO and KEGG databases. We found that these 33 PRGs were mainly involved in the positive regulation of cytokine production, interleukin-1 production, regulation of inflammatory response, pyroptosis, inflammasome complex, cysteine-type endopeptidase activity involved in apoptotic process, cysteine-type endopeptidase activity, and cytokine receptor binding in GO analysis (Fig. 2A). Moreover, KEGG pathway analysis suggested that 33 PRGs were mainly involved in the NOD-like receptor signalling pathway, *salmonelia* infection, cytosolic DNA-sensing pathway, TNF signalling pathway, Toll-like receptor signalling pathway, and apoptosis (Fig. 2B).

### Construction of a pyroptosis-related prognostic gene model

**Fig. 3:**
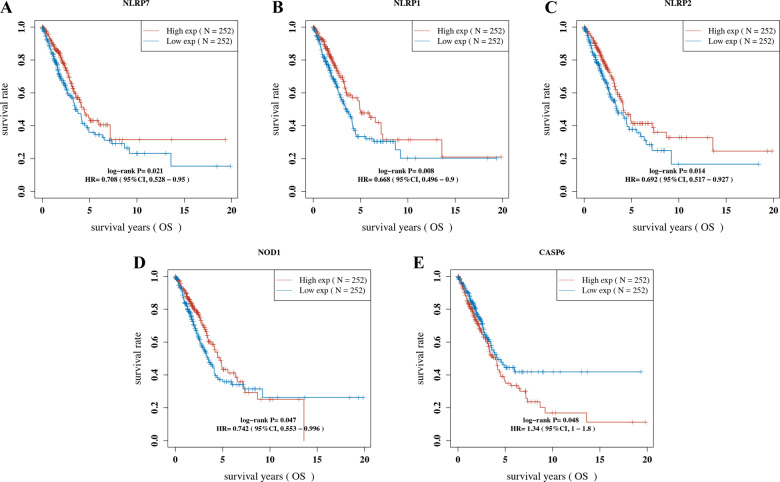
The prognostic value of PRG in LUAD. The overall survival curve of NLRP7 **A** NLRP1 **B** NLRP2 **C** NOD1 **D** and CASP6 **E** in LUAD patients in the high-/low-expression group. PRG pyroptosis‑related gene, LUAD lung adenocarcinoma.

**Fig. 4:**
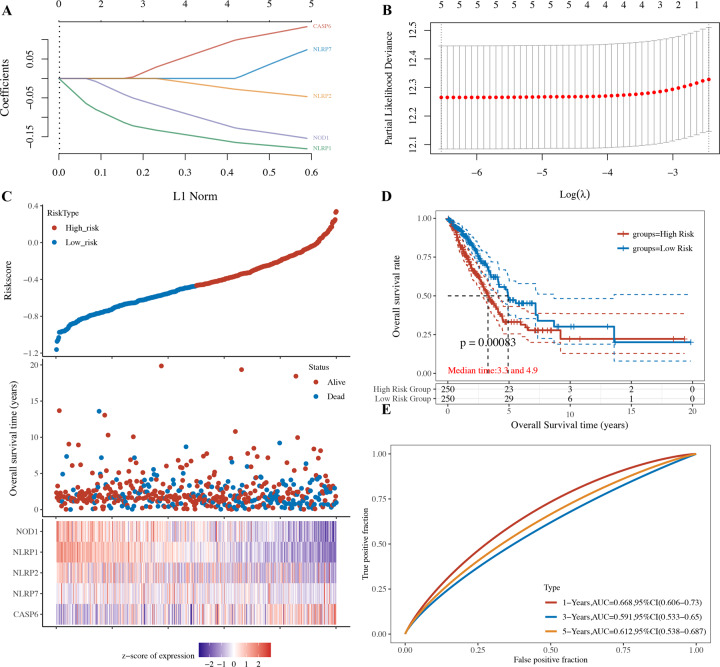
Construction of a prognostic PRG model. **A** LASSO coefficient profiles of the five PRGs. **B** Plots of the ten-fold cross-validation error rates. **C** Distribution of risk score, survival status, and the expression of five prognostic PRGs in LUAD. **D, E** Overall survival curves for LUAD patients in the high-/low-risk group and the ROC curve of measuring the predictive value. PRG, pyroptosis‑related gene; LUAD, lung adenocarcinoma.

To construct a prognostic gene model, univariate Cox regression analysis was performed to screen those PRG with a prognostic value. As a result, a total of five genes with a prognostic value were identified, and the Kaplan–Meier survival curves are shown in Fig. 3. The results suggested a poor survival rate in LUAD patients with low expression of *NLRP7* (Fig. 3A, *p* = 0.021), *NLRP1* (Fig. 3B, *p* = 0.008), *NLRP2* (Fig. 3C, *p* = 0.014), and *NOD1* (Fig. 3D, *p* = 0.047) and high *CASP6* expression (Fig. 3E, *p* = 0.048). LASSO Cox regression analysis was performed to construct a prognostic gene model based on these five prognostic PRGs (Fig. 4A, B). The risk score = (0.0946) * CASP6 + (0.1573) * NLRP7 + (−0.124) * NOD1 + (−0.1627) * NLRP1 + (−0.0262) * NLRP2. Based on the risk score, LUAD patients were separated into two groups. The risk score distribution, survival status, and the expression of these five genes are presented in Fig. 4C. As the risk score increased, the patients’ risk of death increased, and the survival time decreased (Fig. 4C). The Kaplan–Meier curve revealed that LUAD patients with high-risk scores had a worse overall survival probability than those with low-risk scores (median time = 3.3 years vs. 4.9 years, *p* = 0.00083, Fig. 4D), with AUCs of 0.668, 0.591, and 0.612 in the 1-year, 3-year, and 5-year ROC curves, respectively (Fig. 4E).

### Building a predictive nomogram

**Fig. 5:**
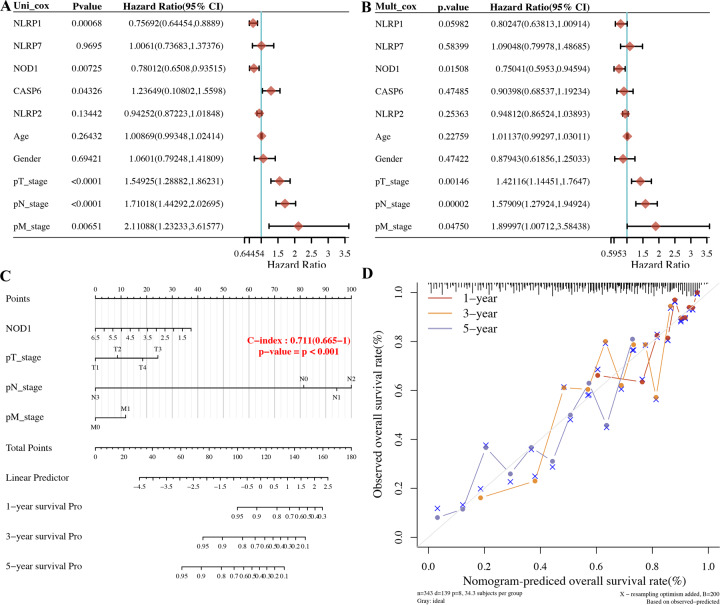
Construction of a predictive nomogram. **A, B** Hazard ratio and *P*‐value of the constituents involved in univariate and multivariate Cox regression considering clinical the parameters and five prognostic PRG in LUAD. **C, D** Nomogram to predict the 1-year, 3-year, and 5-year overall survival rate of LUAD patients. Calibration curve for the overall survival nomogram model in the discovery group. A dashed diagonal line represents the ideal nomogram. PRG pyroptosis‑related gene, LUAD lung adenocarcinoma.

Considering the clinicopathologic features and these five prognostic PRGs, we also built a predictive nomogram to predict the survival probability. Univariate and multivariate analyses revealed that *NOD1* expression and pT stage, pN stage, and pM stage were independent factors affecting the prognosis of LUAD patients (Fig. 5A, B). The predictive nomogram suggested that the 3‐year and 5‐year overall survival rates could be predicted relatively well compared with an ideal model in the entire cohort (Fig. 5C, D).

### PRGs were associated with tumour immune infiltration in LUAD

**Fig. 6:**
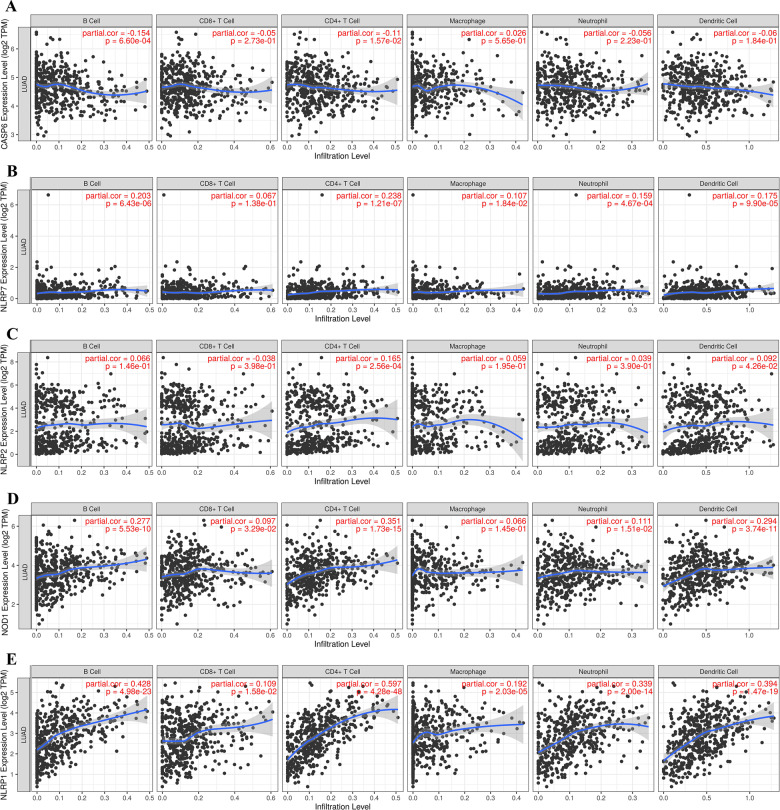
The association between five prognostic PRG and immune infiltration (TIMER). The association between the abundance of immune cells and the expression of CASP6 **A** NLRP7 **B** NLRP2 **C** NOD1 **D** and NLRP1 **E** in LUAD. PRG pyroptosis‑related gene, LUAD lung adenocarcinoma.

Pyroptosis plays a vital role in the development of the tumour-immune microenvironment. In our study, we also clarified the correlation of the expression of prognostic PRGs (*NOD1*, *CASP6*, *NLRP1*, *NLRP2*, and *NLRP7*) and immune infiltration in LUAD using the TIMER database. The data demonstrated a negative correlation between *CASP6* expression and the abundance of B cells (Fig. 6A, *p* = 6.6e^−4^) and CD4 + T cells (Fig. 6A, *p* = 0.0157). Moreover, there was a positive association between *NLRP7* expression and the immune infiltration level of B cells (*p* = 6.43e^−6^), CD4 + T cells (*p* = 1.21e^−7^), macrophages (*p* = 0.0184), neutrophils (*p* = 4.67e^−4^), and dendritic cells (*p* = 9.9e^−5^) (Fig. 6B). *NLRP2* expression showed a positive association with the abundance of CD4 + T cells (*p* = 2.56e^−4^) and dendritic cells (*p* = 0.0426) (Fig. 6C). Figure 6D shows the correlation between *NOD1* expression and the abundance of immune cells, which revealed a positive correlation between *NOD1* expression and the abundance of B cells (*p* = 5.53e^−10^), CD8 + T cells (*p* = 0.0329), CD4 + T cells (*p* = 1.73e^−15^), neutrophils (*p* = 0.0151) and dendritic cells (*p* = 3.74e^−11^). We also found that *NLRP1* expression was positively correlated with the abundance of B cells (*p* = 4.98e^−23^), CD8 + T cells (*p* = 0.0158), CD4 + T cells (*p* = 4.28e^−48^), macrophages (*p* = 2.03e^−5^), neutrophils (*p* = 2e^−14^), and dendritic cells (*p* = 1.47e^−19^) (Fig. 6E). This evidence suggests a significant correlation between PRG and tumour-immune infiltration.

### TMB, MSI, and drug-sensitivity analysis of PRGs

**Fig. 7:**
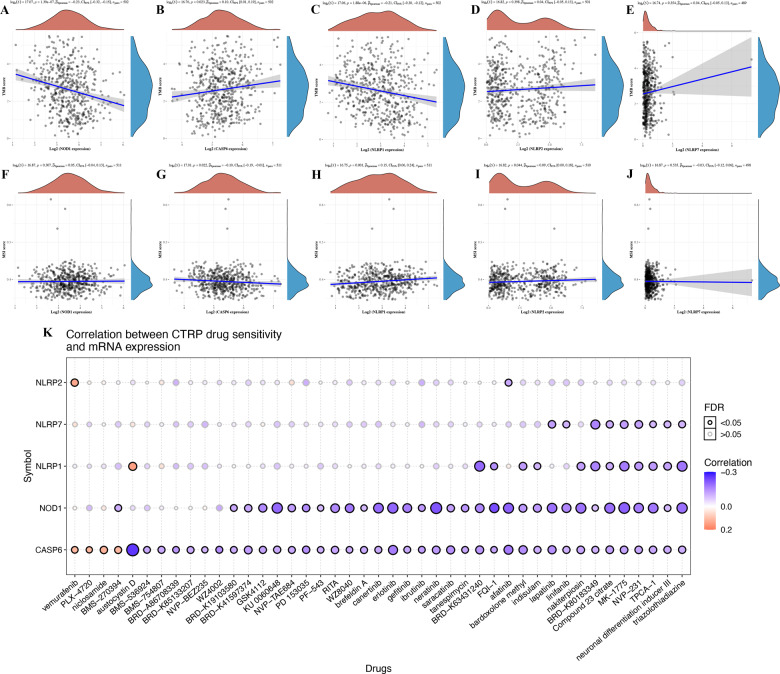
TMB, MSI, and drug-sensitivity analysis of PRG in LUAD. **A–E** The correlation between five prognostic PRG and TMB in LUAD. **F–J** The correlation between five prognostic PRG and MSI in LUAD. **K** The correlation between five prognostic PRG and CTRP drug sensitivity in LUAD. TMB tumour mutation burden, MSI microsatellite instability, LUAD lung adenocarcinoma, PRG pyroptosis‑related gene, CTRP cancer therapeutics response portal.

TMB can be used as a biomarker to predict the efficacy of immunotherapy for lung cancer [12, 13]. Microsatellite instability (MSI) was also suggested as a predictive biomarker for cancer immunotherapy [14]. The above results revealed that the PRG was significantly correlated with tumour immune infiltration. To clarify whether these PRGs could also serve as biomarkers for drug screening, we then analysed the correlation between PRGs and TMB as well as MSI in LUAD. The results revealed a negative correlation between TMB and *NOD1* (Fig. 7A, *p* = 1.39e^−7^) and *NLRP1* (Fig. 7C, *p* = 1.88e^−6^) and a positive correlation between TMB and *CASP6* (Fig. 7B, *p* = 0.023). However, there was no significant correlation between TMB and *NLRP2* and *NLRP7* (Fig. 7D, E). In MSI analysis, MSI was negatively correlated with *CASP6* expression (Fig. 7G, *p* = 0.022), and was positively correlated with the expression of *NLRP1* (Fig. 7H, *p* = 0.001) and *NLRP2* (Fig. 7I, *p* = 0.044). There was no significant correlation between MSI and *NOD1* or *NLRP7* (Fig. 7F, J). To develop a therapy target, it is important to analyse the correlation between gene expression and existing drugs. In our study, drug-sensitivity analysis revealed that the expression of *NLRP7*, *NLRP2*, *NOD1*, and *CASP6* was positively correlated with some or most drugs in the cancer therapeutic response portal database (Fig. 7K).

### Construction of a network of mRNA–miRNA–lncRNA

**Fig. 8:**
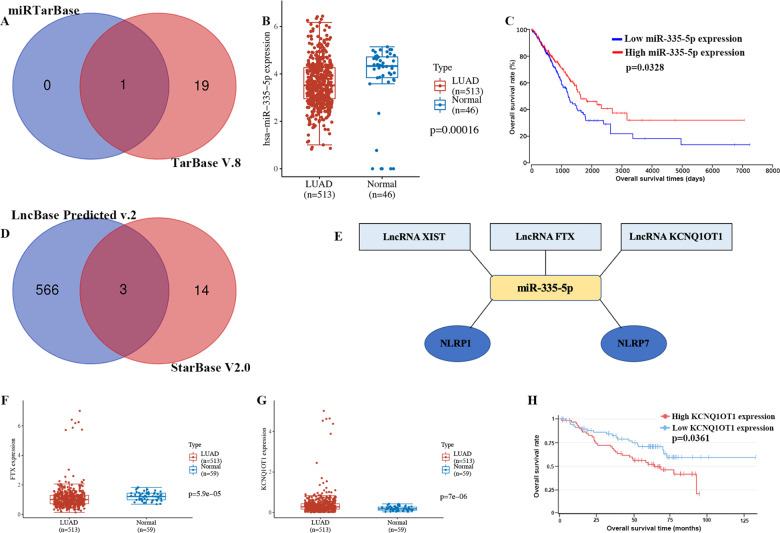
Construction of ceRNA network. A Results of miRNA target predicted by mirTarBase and TarBase V.8. The expression **B** and prognostic value **C** of miR-335-5p in LUAD. **D** Results of lncRNA targets predicted by lncBase predicted V.2 and StarBase V2.0. **E** The network of lncRNA–miRNA–mRNA. **F-G** The expression lncRNA FTX and lncRNA KCNQ1OT1 in LUAD. **H** The prognosis value of lncRNA KCNQ1OT1 in LUAD. LUAD lung adenocarcinoma; CeRNA competing endogenous RNA.

We also clarified the correlation between prognostic PRG and clinical stage, which revealed that *NLRP1* (Fig. S2C, *p* = 0.0105) and *NLRP7* (Fig. S2E, *p* = 0.00211) were correlated with clinical stage. However, there was no significant correlation between *NOD1* (Fig. S2A, *p* = 0.339), *CASP6* (Fig. S2B, *p* = 0.232) and *NLRP2* (Fig. S2D, *p* = 0.645), and clinical stage. This suggested that *NLRP1* and *NLRP7* may be involved in tumour progression in LUAD. To clarify the potential molecular mechanism of *NLRP1* and *NLRP7* in LUAD, we then constructed a network of mRNA–miRNA–lncRNA interactions. The data identified miR-335-5p as the targeting mRNA binding to *NLRP1* and *NLRP7* according to mirTarBase and TarBase V.8 (Fig. 8A). Further analysis revealed that miR-335-5p was downregulated in LUAD (Fig. 8B, *p* = 0.00016), and LUAD patients with high miR-335-5p levels experienced better overall survival (Fig. 8C, *p* = 0.0328). According to this result, we also explored its upstream lncRNA targets to construct the miRNA–lncRNA axis. As shown in Fig. 8D, three lncRNAs, lncRNA XIST, lncRNA FTX, and lncRNA KCNQ1OT1, were identified as targets. The ceRNA network is shown in Fig. 8E. The expression of lncRNA targets was also detected, which revealed downregulation of lncRNA FTX (Fig. 8F, *p* = 5.9e^−5^) and upregulation of lncRNA KCNQ1OT1 (Fig. 8G, *p* = 7e^−6^) in LUAD compared with normal tissues. However, only lncRNA KCNQ1OT1 could reduce the LUAD patients’ survival probability (Fig. 8H, *p* = 0.0361). Thus, the lncRNA KCNQ1OT1/miR-335-5p/NLRP1/NLRP7 regulatory axis may play a vital role in the progression of LUAD.

## Discussion

Pyroptosis is a newly recognised type of programmed cell death that exerts a dual function in cancer progression and treatment mechanisms. Pyroptosis can release inflammatory factors and stimulate normal cells, resulting in transformation into tumour cells [15]. However, pyroptosis can promote tumour cell death, making pyrolysis a potential prognostic and therapeutic target for cancer [16]. In ovarian cancer, a novel PRG signature has been identified to predict prognosis [10]. However, the role of PRG in LUAD has not yet been elucidated, and our study was performed to clarify this role.

We first clarified the expression and prognostic value of PRGs in LUAD. We found that the expression of *PRKACA*, *NOD1*, *NLRP1*, *ELANE*, *TNF*, *IL1B*, *IL18*, *PYCARD*, *CASP5*, *NLRC4*, *NLRP3*, *IL6*, and *CASP1* was increased, while the expression of *GSDMB*, *PJVK*, *CASP4*, *NLRP7*, *CASP3*, *CASP6*, *CASP8*, *GSDMA*, *GSDMC*, and *AIM2* was decreased in LUAD compared with normal tissues. Prognosis analysis suggested a poor survival rate in LUAD patients with low expression of *NLRP7*, *NLRP1*, *NLRP2*, and *NOD1* and high *CASP6* expression. These data were consistent with prior results. Edward et al. suggested that low expression of *NLRP1* was linked to a poor prognosis and immune infiltration in LUAD [17].

We also performed functional enrichment analysis of PRGs, which revealed that these 33 PRGs were mainly involved in the regulation of the inflammatory response, pyroptosis, NOD-like receptor signalling pathway, TNF signalling pathway, Toll-like receptor signalling pathway, and apoptosis. Interestingly, these functions or pathways were correlated with the oncogenesis and progression of LUAD. The induction of Th1-like and cytotoxic immunity by the TLR signalling pathway could result in lung cancer regression or arrest [18]. Moreover, a previous study showed that potentially functional genetic variants in TNF/TNFR signalling pathway genes were associated with prognosis in LUAD [19]. These results suggested that these 33 PRGs may also play a vital role in the oncogenesis and progression of LUAD.

LASSO Cox regression analysis was performed to construct a prognostic gene model based on five prognostic PRGs (*NLRP7*, *NLRP1*, *NLRP2*, *NOD1*, and *CASP6*), which could predict the overall survival of LUAD patients with medium-to-high accuracy. A predictive nomogram suggested that the 3‐year and 5‐year overall survival rates could be predicted relatively well compared with an ideal model in the entire cohort. A previous study identified several prognostic signatures for LUAD. A study performed by Sijin developed and validated an immune-related prognostic signature in LUAD [20]. Another glycolysis-related gene signature could predict metastasis and survival in LUAD patients [21]. Moreover, an autophagy-related prognostic signature showed good performance in LUAD patient prognosis prediction [22]. In our study, we first identified a pyroptosis-related prognostic gene signature for LUAD, which provides more choices for prognostic prediction in LUAD.

In our study, *CASP6* was found to be one of the gene signatures. Although a previous study revealed that CASP6 could facilitate the activation of programmed cell death pathways, including pyroptosis, apoptosis, and necroptosis, CASP6 is typically not associated with pyroptosis [23]. CASP6 is generally considered to be a vital regulator of innate immunity, inflammasome activation, and host defence [23]. Increasing evidence has revealed that CASP6 is involved in carcinogenesis and progression by regulating the apoptosis and metastasis of tumours [24]. Moreover, active CASP6 is thought to be a potential therapeutic target against Alzheimer’s disease [25, 26]. This combined evidence suggested a broad role for CASP6. However, studies on the role of CASP6 in pyroptosis are limited. In our study, we found that *CASP6* was one of the pyroptosis-related prognostic biomarkers in LUAD. Further in vivo and in vitro studies should be performed to verify whether CASP6 is involved in pyroptosis in LUAD.

Another important finding of our study revealed that the above five pyroptosis-related prognostic genes were significantly correlated with immune infiltration, which further confirmed the fact that pyroptosis plays a vital role in the tumour immune microenvironment. *BRAF* mutations could regulate the tumour immune microenvironment by regulating the pyroptosis-related signalling pathway [27]. A previous study also found that the pyroptosis gene *NLRP1* is correlated with immune infiltration in LUAD [17].

We also constructed a mRNA–miRNA–lncRNA network, which identified a lncRNA KCNQ1OT1/miR-335-5p/NLRP1/NLRP7 regulatory axis. In fact, miR-335-5p could regulate the LUAD cell cycle and metastasis [28]. Moreover, miR-335-5p could suppress TGF-β1-induced EMT in lung cancer [29]. Interestingly, lncRNA KCNQ1OT1 could accelerate LUAD cell proliferation, migration, and invasion [30]. In our study, we also found that miR-335-5p and lncRNA KCNQ1OT1 were linked to the prognosis of LUAD patients. All this evidence suggests that the lncRNA KCNQ1OT1/miR-335-5p/NLRP1/NLRP7 regulatory axis may also play an important role in the progression of LUAD. Further study should be conducted to verify this result.

Our study has some limitations. All analyses were conducted using the TCGA LUAD cohort, and it would be better to verify them using the GEO cohort. Moreover, in vivo and in vitro experiments should be performed to further confirm our results.

In conclusion, we performed a comprehensive and systematic bioinformatics analysis and identified the pyroptosis‑related prognostic gene signature containing five genes (*NLRP7*, *NLRP1*, *NLRP2*, *NOD1*, and *CASP6*) for LUAD patients. Our results also identified a lncRNA KCNQ1OT1/miR-335-5p/NLRP1/NLRP7 regulatory axis, which may also play an important role in the progression of LUAD. Further study should be conducted to verify this result.

## Materials and methods

### Datasets and preprocessing

The RNA-sequencing (RNA-seq) data of 486 LUAD patients and the corresponding clinical information were obtained using The Cancer Genome Atlas (TCGA) database on April 1, 2021. The clinical information of the LUAD patients is shown in Table S1. Moreover, somatic datasets and copy number variation (CNV) data for LUAD were also downloaded from TCGA and the University of California, Santa Cruz (UCSC) Xena website, respectively. Data analysis was performed with the R (version 4.0.5) and R Bioconductor packages. The expression data were normalised to transcripts per kilobase million (TPM) values before further analysis.

### Identification of differentially expressed PRGs

A total of 33 PRGs were obtained from prior reviews [10, 31], which are shown in Table S2. The difference in PRG expression in LUAD and normal tissues was identified using the “limma” and “reshape2” packages. We then constructed a protein–protein interaction (PPI) network for 33 PRGs using the Search Tool for the Retrieval of Interacting Genes (STRING).

### Mutation analysis of PRGs

The mutation frequency and oncoplot waterfall plot of 33 PRGs in LUAD patients were generated by the “maftools” package. The location of CNV alteration of 33 PRGs on 23 chromosomes was drawn using the “RCircos” package in R.

### Functional enrichment analysis

Gene Ontology (GO), including the biological process (BP), cellular component (CC), and molecular function (MF) categories, was conducted with the “ggplot2” package in R software. Similarly, this package was also utilised to perform Kyoto Encyclopedia of Genes and Genomes (KEGG) analysis.

### Development of the pyroptosis-related gene prognostic model

Cox regression analysis was performed to evaluate the prognostic significance of the PRGs. For Kaplan–Meier curves, *p*-values and hazard ratios (HRs) with 95% confidence intervals (CIs) were generated by log-rank tests and univariate Cox proportional hazard regression. PRGs with a significant prognostic value were selected for further analysis. Based on these prognostic PRGs, LASSO Cox regression analysis was then used to construct the prognostic model. The TCGA LUAD patients were divided into low- and high-risk subgroups according to the median risk score, and the overall survival (OS) time was compared between the two subgroups via Kaplan–Meier analysis. The predictive accuracy of each gene and the risk score were evaluated by performing time receiver-operating characteristic (ROC) analysis. Considering the clinical characteristics, a predicted nomogram was developed to predict the 1-, 3-, and 5-year overall survival. A forest was used to show the *P*-value, HR and 95% CI of each variable through the “forestplot” R package.

### Immune infiltration, tumour mutation burden, and microsatellite-instability analysis

We then analysed the correlation between prognostic PRG and immune infiltration using the Tumour IMmune Estimation Resource (TIMER, https://cistrome.shinyapps.io/timer/), a web portal for comprehensive analysis of tumour-infiltrating immune cells. The “Gene” module of TIMER could visualise the correlation of gene expression with the immune infiltration level in LUAD. In tumour mutation burden (TMB) and microsatellite-instability (MSI) analysis, Spearman’s correlation analysis was performed to calculate the correlation between gene expression and TMB and MSI score. A p-value of less than 0.05 was considered statistically significant.

### Competing endogenous RNA network construction

To clarify the potential function of PRG in LUAD, we then constructed a competing endogenous RNA (ceRNA) network. miRTarBase (http://mirtarbase.cuhk.edu.cn/) and TarBase V.8 (https://carolina.imis.athena-innovation.gr/diana_tools/web/index.php?r=tarbasev8%2Findex) were utilised to predict the miRNA targets binding to the PRGs. Based on the miRNAs identified, StarBase (http://starbase.sysu.edu.cn/) and LncBase Predicted v.2 (https://carolina.imis.athena-innovation.gr/diana_tools/web/index.php?r=lncbasev2/index-predicted) were utilised to predict lncRNA targets interacting with miRNAs. We also explored the expression and prognostic value of these miRNA and lncRNA targets using the TCGA LUAD dataset. All analyses were considered statistically significant at *P* < 0.05.

## Supplementary information

Supplementary Table

Supplementary Figure 1

Supplementary Figure 2

## Data Availability

The analysed data sets generated during the study are available from the corresponding author on reasonable request.
