# Supplementary figures and images for: Identification of the pyroptosis‑related prognostic gene signature and the associated regulation axis in lung adenocarcinoma
Source: Cell Death Discov. 2021 Jun 25;7:161. doi: 10.1038/s41420-021-00557-2 (PMC8257680; doi:10.1038/s41420-021-00557-2)

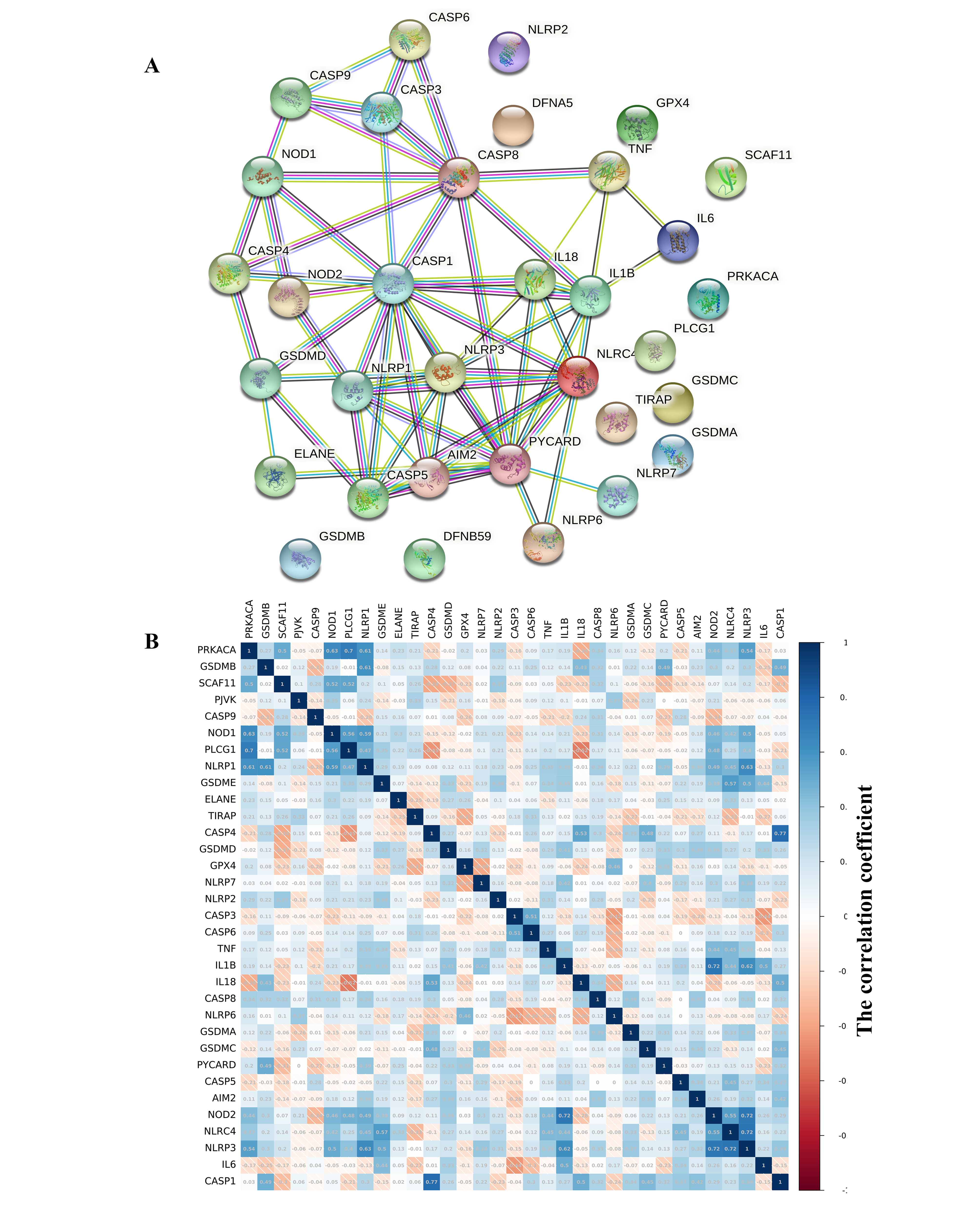

Supplement: Supplementary file 2 — Supplementary Figure 1 [file 41420_2021_557_MOESM2_ESM.tif]

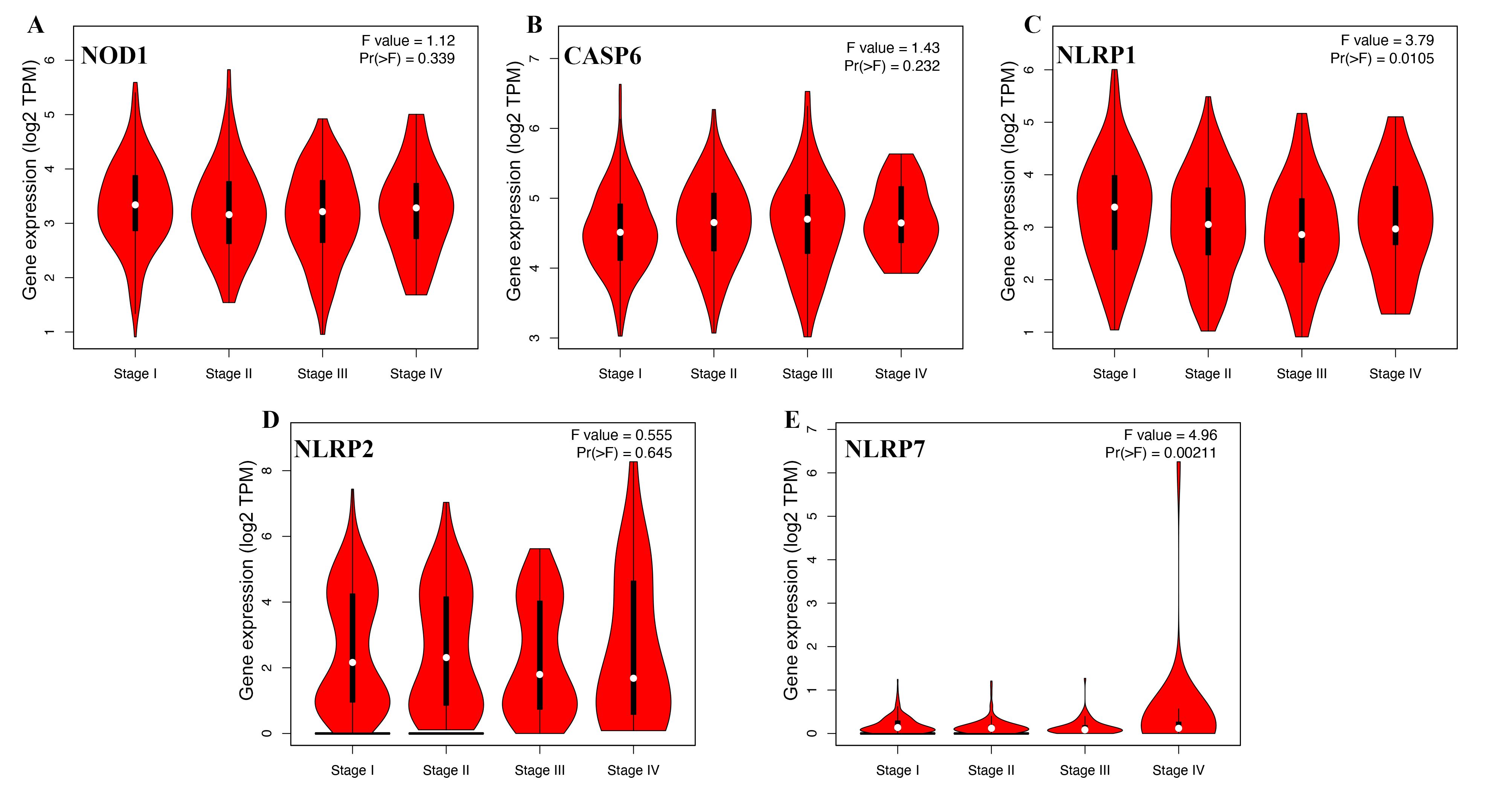

Supplement: Supplementary file 3 — Supplementary Figure 2 [file 41420_2021_557_MOESM3_ESM.tif]
